# Existential Well-being, Mental Health, and COVID-19: Reconsidering the Impact of Lockdown Stressors in Moscow

**DOI:** 10.11621/pir.2022.0202

**Published:** 2022-06-15

**Authors:** Anastasia Y. Klimochkina, Elena V. Nekhorosheva, Daria A. Kasatkina

**Affiliations:** a HSE University, Moscow, Russia; b Moscow City University, Moscow, Russia

**Keywords:** Well-being, existential psychology, COVID-19 pandemic in Russia, COVID-19-related stressors, mental health, existential fulfillment

## Abstract

**Background:**

Initial psychological papers on COVID-19, mental health and wellbeing mostly focus on the aftermath lockdown-related stress and stress related to the disease itself. Still, we presume that personal well-being can be resistant to stressors depending on the way the person is settled in their life.

**Objective:**

We seek to reconsider the contribution of lockdown-related stressors to existential well-being, to assess existential well-being during the outbreak and to compare the contribution of living conditions and COVID-19-related factors on well-being.

**Design:**

An online survey was conducted during the peak of the outbreak in Moscow (April-May 2020) (N=880). The data was obtained using the “Test of Existential Motivations” questionnaire and a series of questions addressing (1) living conditions — mental and physical health, employment, and social distancing; (2) COVID-19-related stressors — non-chronic illness, financial losses, and unavailability of goods or services; (3) sociodemographic indicators — age, gender, and income. Data analysis included hierarchical multiple regression, one-sample t-test, and analysis of variance.

**Results:**

Surprisingly, the existential well-being of Moscow citizens during the research period was moderate. Each of the three groups of factors predicted a similar proportion of the variance of well-being (3-3,9%). The strongest predictors of well-being were long-term mental health status and financial stability. The effect of COVID-19-related stressors was most pronounced when they co-occur.

**Conclusion:**

The negative association between lockdown-related stressors and poor well-being is not universal. It is necessary to study the effect of COVID-19-related stressors in combination with individual living conditions and region-specific factors and to focus on the prevention of the occurrence of stressors.

## Introduction

The COVID-19 pandemic generated social and psychological changes globally. Mental health specialists have been registering various behavioral and psychological challenges, such as hoarding behavior, emotional eating, dependencies, anxiety, and depression (Banerjee, 2020; Barcın-Güzeldere, 2022; Rajkumar, 2020; Talevi et al., 2020; Zandifar & Badrfam, 2020). The negative impact of the pandemic on mental health was observed during various “waves” of the pandemic and was considered more harmful than other stressful events (Olff et al., 2021). Some researchers noticed a delayed or cumulative effect of the pandemic on people’s well-being (Zacher and Rudolph, 2020).

Many scientists have stated that the COVID-19 pandemic, lockdown, and situational factors such as harsh security measures, self-isolation, fear of being infected, a lack of relevant information, loneliness, boredom, and financial troubles, negatively affected people’s mental health (Capuzzi et al., 2020; Newby et al., 2020; Rajkumar, 2020; Satici et al., 2020; Tian et al., 2020; Yıldırım et al., 2020). Though many scholars observed similar mental health issues across different countries, the effects of lockdown on well-being differed. Ausín et al. (2021), comparing Spanish and Russian general populations, stated that loneliness and alienation, as a tendency to gain social support from family only, were more pronounced among the Russian population.

Some researchers have highlighted that a person’s lifestyle and level of life satisfaction before the onset of COVID-19 could predict how they would feel during the pandemic (Hoffman, 2020; Trzebiński et al., 2020; Yang, 2020). Sutin et al. (2020) noted that people remain resilient in the face of catastrophic events despite the stress they cause, at least in the short term.

We aim to reconsider the contribution of lockdown-related stressors to the level of existential well-being. Our goal was to compare the contributions of long-term and short-term (lockdown-related) factors affecting self-reported existential well-being, and to assess the level of Muscovites’ well-being during the most stressful period of the pandemic. We hypothesized that, despite initial studies of the psychological effects of the pandemic, long-term factors were more significant for existential wellbeing than short-term stressors and that the well-being of the participants would not be poor.

### COVID-19 in Russia: Background

COVID-19 began to spread in Russia at the end of January 2020 (Mankoff, 2020). Lockdown restrictions varied from region to region and according to morbidity levels. In Moscow, which has a registered population of around 12.5 million, a high-alert regime was imposed on March 5^th^, while the morbidity was still low (Moscow Government, 2020a). Moscow residents were obliged to inform the authorities of their condition and self-isolate for 14 days after returning from abroad. The authorities canceled all public events with over 5,000 participants. By March 12^th^, there were 25 new cases in Moscow and the Moscow Region, compared with 45,000 cases with 4,917 deaths worldwide.

A strict lockdown was introduced in Moscow on April 15^th^ (Moscow Government, 2020b). Following this, residents were required to stay at home or use a digital pass for any travel. A shortage problem and a temporary price increase occurred for certain goods, including medicines, medical masks, and antiseptics. Temporary hospitals were opened. Students began to study online.

Morbidity reached its height by May 7^th^, with 6,703 new cases and 39 deaths in Moscow and 842 new cases and 15 deaths in the Moscow Region. Moscow authorities introduced one-time payments to support families with children, pensioners, and the unemployed and provided a COVID-19 hotline on the Moscow Mayor’s official webpage. Most employees started to work remotely (Nekhorosheva et al., 2020), and business tax holidays were introduced.

By June 9^th^, the morbidity level in Moscow decreased to 1,500 new cases and 12 deaths, compared with 7.3 million global cases, with a daily increase of 124,700 cases and 32,474 deaths. Moscow authorities gradually put an end to lockdown restrictions and canceled digital passes, many small businesses and services reopened.

### Understanding Mental Health and Well-being: An Existential Approach

Well-being is a core concept in mental health science. According to the World Health Organization (WHO, 2001, 2018), mental health is both absence of mental disorders and a state of emotional, mental, and behavioral well-being that allows for adaptation to everyday life. Mental health implies the ability to deal with the stressors of daily life, fulfill one’s potential, and work fruitfully (WHO, 2013). The pandemic has drastically altered several societal fundamentals, such as security of life, reliability of public institutions, and freedom of movement and communication. We take an existential approach to assessing these fundamental changes. Existential models allow us to consider the psychological characteristics of a person’s quality of life by assessing their interaction with external life circumstances (Längle, 1993; Längle & Klaassen, 2019).

Anxiety about one’s mortality (death anxiety) is a fundamental concept in existential psychology (Frankl, 1992; Yalom, 1980, 2008), which is thrust to the forefront of our minds due to awareness of the threat posed by the virus. For example, Tomaszek and Muchacka-Cymerman (2020) studied the mediating effect of existential anxiety and life satisfaction on the relationship between PTSD symptoms and post-traumatic growth during the pandemic. Existential psychology understands wellbeing as fulfillment, perceiving life as good, having inner consent to life’s conditions and limitations, and choosing an authentic way of life (Längle, 2003). An existentially prosperous person can cope with everyday tasks, build warm relations with themselves and others, have healthy emotions, be authentic and productive, and make meaningful contributions to the future (Längle, 2011, 2014).

### Theoretical Model: Three Groups of Factors Impacting Existential Well-being During COVID-19

Applying an existential approach, we identified three groups of factors that could predict the psychological fallout of lockdown.

The first group includes the sociodemographic features that influence social status and living conditions — age, gender, monthly household income. According to researchers, females, children, adolescents, and the elderly are prone to anxious or depressive reactions during the pandemic (Brooks et al., 2020; Fernández-Castillo et al., 2021; Inchausti et al., 2020; Rajkumar, 2020; Yenan Wang et al., 2020). Women who have experienced traumatic events are more likely to develop anxiety symptoms (Cai et al., 2021; Remes et al., 2016). Women and the elderly were more open to help-seeking behavior (MacKenzie et al., 2008; Mojtabai et al., 2002). Researchers stress the differences in “socially acceptable methods of coping with stress and care-seeking rates for mental disorders between men and women” (Cabrera-Mendoza et al., 2020, p. 68). People with lower incomes could suffer from fear and stigmatization (Tian et al., 2020). All this justifies the inclusion of this group of factors into the model as control variables.

The second group refers to ongoing living conditions and individual way of life. It includes (1) mental health status (diagnosed psychiatric conditions such as depression and anxiety), (2) physical health status (chronic physical conditions such as hypertension, lung disease, and heart disease), (3) working status (employment of any type, or non-working status including being a housewife, student, or pensioner), and (4) self-isolation or social distancing (the degree of changes in personal daily life and behavior caused by the lockdown restrictions).

Tian et al. (2020) demonstrated that employment, financial problems, lower levels of education, and migrant status had affected the mental health of Chinese citizens, while mental health literacy among Chinese college students was associated with lower stress and anxiety levels (Hu et al., 2021). The impact of poor health conditions, specifically diagnosed mental disorders, has been noted in COVID-19 studies in different countries (Newby et al., 2020). The relevance of self-isolation behavior has also been widely discussed (Rubin & Wessely, 2020; Taylor, 2019). For example, Talevi et al. (2020) found that increased length and severity of quarantine was associated with increased anxiety, depression, coping strategies, and stigmatization.

Situational stressors constitute the third group of factors in our model. *A psychological stressor* is a “life situation that creates an unusual or intense level of stress that may contribute to the development or aggravation of mental disorders, illness, or maladaptive behavior” (VandenBos, 2015, p. 1204). We study the following situational COVID-19-related stressors: health, financial complications, and lockdown.

The first stressor is becoming infected with COVID-19 or having a family member infected. Fear of death, loss of loved ones, damage to health, and lack of information (the so-called “headline stress disorders’’) can provoke a stress reaction. People diagnosed with COVID-19 experienced different mental outcomes depending on the severity of the disease and quarantine conditions — from anxiety, shame, and stigmatization (Tian et al., 2020), to post-traumatic stress symptoms (Bo et al., 2020). People who did not suffer COVID-19 experienced the emergence of defensive mechanisms, panic, and various anxiety-related reactions due to abundant or controversial reports about regarding virus and the epidemiological situation (Cuiyan Wang et al., 2020; Dong & Zheng, 2020; Zandifar & Badrfam, 2020).

The second stressor is the economic crisis which creates financial losses, unemployment, and unpredictability. This stressor leads to social fears, xenophobia, detachment, anxiety, and depressive disorders (Banerjee, 2020; Talevi et al., 2020).

The third stressor is the lockdown itself, manifested in restrictions, loss of freedom, social distancing, lack of social contacts, routine changes, and inaccessibility of some basic supplies. It triggers various feelings (anger, irritation, confusion, anxiety, loneliness), post-traumatic stress symptoms, and other severe psychological and behavioral deviations, such as suicidality, dependencies, and somatization (Banerjee, 2020; Bo et al., 2020; Brooks et al., 2020; Inchausti et al., 2020; Roy et al., 2020; Talevi et al., 2020; Yenan Wang et al., 2020).

## Methods

This study aims to assess the existential well-being of Muscovites during the lockdown period and compare the contribution of the participants’ living conditions and COVID-19-related factors on well-being. We hypothesized that factors relating to long-term and ongoing life events would have a more significant impact on existential well-being than short-term stressors and that the participants’ well-being would not be poor.

In order to test this hypothesis, we compared the unique contributions of two groups of factors (ongoing living conditions and situational COVID-19-related stressors) towards levels of existential well-being, while controlling sociodemographic variables. The factors were structured so as to compare the relevance of long-term dispositions and short-term stressors in the same areas of life: (1) health, (2) work, and (3) state of social distancing during the pandemic. Each factor is treated as an independent variable, while the dependent variable is existential fulfillment as a measure of well-being (Shumskiy et al., 2017; Shumskiy & Klimochkina, 2018).

We used a cross-sectional research design. The quantitative data was collected using verbal questionnaires based on self-reports. The survey was conducted online due to lockdown restrictions.

### Participants

The raw sample consisted of 1839 unique answers, before the following exclusion criteria were applied:

1. Agreement for the processing of personal data.
2. No missing data (all fields were filled).
3. Using the answer “prefer not to say” in the question about monthly family income.

The final sample consisted of 880 participants (9.2% male, 90.8% female; M_age_ = 39.55 years, SD = 10.33, range = 17–75 years) (see *Table 1*).

**Table 1:** Sample characteristics (N=880)

	Characteristics	Frequency	Valid Percent
Socio-demographic characteristics			
Age	17–20	36	4%
	21–30	112	13%
	31–40	320	36%
	41–50	303	34%
	51–60	86	10%
	61–75	23	3%
Gender	male	81	9%
	female	799	91%
Household income per month	subsistence level (< 50000 RUB)	260	30%
	middle level (50000–100000 RUB)	418	48%
	prosperous level (> 100000 RUB)	202	23%
Ongoing life situation			
Mental health status	none	849	96%
	neuropsychiatric conditions	31	4%
Somatic health status	none	591	67%
	chronic somatic conditions	289	33%
Working status	unemployed/non-working citizens (including social categories of citizens — students, pensioners, persons with dis-abilities)	267	30%
	employed/working citizens	613	70%
Social distancing	strict self-isolation	72	8%
	reasonable self-isolation	596	68%
	no self-isolation / partial social distancing	212	24%
Situational stressors			
Disease	none	821	93%
	faced any disease himself or a family member	59	7%
Financial difficulties	none	613	70%
	faced earnings restrictions or job loss	267	30%
Lockdown difficulties	none	523	59%
	faced unavailability of goods, medicines, or services	357	41%

The average monthly family income (50 000–100 000 RUB^1^) was reported by 47.5% of respondents, 29.5% reported a subsistence level of income for a two-person family living in Moscow (< 50 000 RUB), 23% had a high level of income (> 100 000 RUB). The respondents exhibited good health: only 4% had been diagnosed with neuropsychological conditions (depression, anxiety, or other) at the time of the survey; 33% had chronic physical conditions (heart disease, lung disease, or other). Concerning working status, most were employed (70%), while 30% were non-working, including housewives, students, pensioners, and persons with disabilities. As for self-isolation status, 68% maintained a reasonable degree of self-isolation, 8% supported all restrictive prescriptions, and 24% reported they had not changed their routine during the pandemic. Respondents faced the following COVID-19-related stressors: 3% fell ill themselves (any infection) or had a family member fal ill; 30% faced a decrease in earnings or job loss; 41% faced the unavailability of goods, medicines, or services during the lockdown.

### Procedure

The study was approved by the Psychological and Pedagogical Research Ethics Committee (PPREC) of the Institute of Pedagogy and Psychology of Education (Moscow City University) on 01/04/2020. The online questionnaire was made on the Survey-Monkey platform. The participants were provided with the web-link sent through urban parental and professional communities (such as academic, pedagogical, medical, and law enforcement communities) using social networks and messengers. Participation in this study was voluntary and anonymous. Participants were also asked to provide electronic consent for the processing of personal data.

The target sample was used, in accordance with the target audience — Muscovites (“living in Moscow”). According to the Federal State Statistics Service, 12.6 million people were living in Moscow by 2020. With the sample reliability of 99%, our sample size (*N=*880) was sufficient. Also, it was important to represent people of different gender, age, occupation, and social status, as well as to comprehensively cover the working part of the city’s population, since changes in the working status and income were expected to be one of the consequences of the pandemic.

The survey was conducted from April 19^th^ to May 18^th^, during the time when citizens were obliged to use digital passes, avoid public places (including schools and kindergartens), wear medical masks, and maintain self-isolation. Most of the data was collected during the first COVID-19 wave in Moscow (from April 27^th^ to May 3^rd^), when restrictions were tightest.

### Statistical analysis

We used R-studio and SPSS software to perform the statistical analysis:

1. One-sample t-test to examine the difference between the sample mean and the standard TEM values for the Russian population.
2. Hierarchical multiple regression to explore the relationship between existential well-being as a dependent variable and the three groups of independent variables: (1) sociodemographic indicators (as controlled variables), (2) ongoing life conditions, and (3) COVID-19-related stressors. Variables were included in each of the groups of factors in accordance with the theoretical model. This analysis allowed us to measure the contribution of COVID-19-related stressors against the long-term living conditions of the respondents. Thus, we could test the claim of whether COVID-19-related stressors had a universally harmful effect, and identify the stressors to which respondents were most sensitive.
3. ANOVA was used to further refine the relationship between categorical variables (and their interactions) and existential well-being.

### Questionnaires

*Existential well-being* was measured using the Test of Existential Motivations questionnaire (TEM) (Shumskiy et al., 2017) based on Längle’s theory of four fundamental existential motivations (Längle, 2016). The questionnaire consisted of 36 items (24 were reverse-scored and 12 straight), 4 subscales (with 9 items in each scale), and one summarizing indicator. Each item was assessed using a Likert scale over a range of 1 to 4, where 1 = “*strongly disagree*”, 2 = “*disagree*”, 3 = “*agree*”, and 4 = “*strongly agree*”. Each subscale represented the prerequisites for existential fulfillment — fundamental motivations (FM): 1 FM referred to fundamental trust; 2 FM referred to the fundamental value of life; 3 FM referred to the authenticity and fundamental self-value, and 4 FM referred to the meaning of life. Due to the need for further confirmation of the factor structure of the questionnaire subscales, this study used only an aggregated indicator of existential well-being.

Other variables were evaluated by direct questions:

1. *Sociodemographic characteristics* were assessed using questions on matters of gender, age, and family monthly income. The option “prefer not to say” was available for the question regarding income.
2. *Ongoing life conditions* were measured using questions concerning:
  - Mental health status: “Do you have any clinically diagnosed mental disorders, such as depression, anxiety disorder, or other clinically diagnosed mental disorder?” (1 = *yes*, 0 = *no*).
  - Physical health status: “Do you have any clinically diagnosed chronic physical disorders, such as hypertonic disease, diabetes, heart diseases, lung disease (including asthma, COPD, etc.), oncological diseases, disability, or mobility limitation, or other clinically diagnosed chronic diseases or vulnerable states?” (1 = *yes*, 0 = *no*).
  - Working status: “Are you currently employed?” (1 = *yes*, 0 = *no*).
  - Social distancing status: “How would you describe your current routine during lockdown?” Respondents were asked to choose from three options: (1) “I am on strict self-isolation or quarantine, I don’t leave home and follow all the authorities’ guidance”; (2) “I can leave home if necessary, following authorities’ guidance on self-isolation and social distancing”; (3) “I am moving freely around the city, and nothing has changed in my daily routine”.
3. *Situational COVID-19-related stressors* were measured with the question: “For the last seven days, have you experienced any of the following?”: (1) “I or my family members have become ill (any illness) and/or had to see a doctor” (1 = *yes*, 0 = *no*); (2) “financial loss, a reduction in earnings or job loss” (1 = *yes*, 0 = *no*); (3) “the unavailability of goods, medicines or services” (1 = *yes*, 0 = *no*).

## Results

We used a one-sample t-test to examine the difference between the sample mean and the value established by the norms of the TEM test for the total Russian population, including Moscow (see *Table 2*).

**Table 2:** Summary of One Sample T-Test for the Level of Existential Well-being (fulfillment)

	General population sample (standard values TEM)		Moscow during Covid-19 sample		t (879)	**Cohen’s** *d*
	M	SD	M	SD		
Existential (fulfillment) well-being	102.8	17.2	106.7	14.8	7.38	0.249
N	923		880			

Note. *** p < 0.00

The mean in the Moscow sample during the lockdown period turned out to be significantly higher than TEM norms. Although the difference was significant, its effect size was relatively small (Cohen’s *d* = 0.249).

We used hierarchical multiple regression to explore the relationship between existential well-being as a dependent variable and the three groups of independent variables (see *Table 3*).

**Table 3:** Summary of Hierarchical Regression Analysis Estimating the Level of Existential Well-being (N = 880)

	Model 1			Model 2			Model 3			Model 4		
	b	SE(b)	β	b	SE(b)	β	b	SE(b)	β	b	SE(b)	β
(Intercept)	91.95***	3.84		92.02***	3.89		97.67***	4.30		96.85***	3.92	
Age	0.14**	0.05	.10	0.13*	0.05	.09	0.10*	0.05	.07	0.10*	0.05	.07
Gender ^a^	3.22	1.70	.06	3.28	1.69	.06	2.60	1.66	.05	2.55	1.66	.05
Income: 1/0 ^b^	3.01**	1.15	.10	2.03	1.16	.07	1.23	1.16	.04	0.97	1.16	.03
Income: 2/0 ^b^	6.55***	1.37	.19	5.11***	1.39	.15	4.03**	1.38	.11	3.99**	1.37	.11
Mental health status ^c^				–13.16***	2.69	–.16	–12.94***	2.65	–.16	–13.10***	2.65	–.16
Somatic health status ^c^				–2.24*	1.14	–.07	–2.14	1.12	–.07	–2.06	1.12	–.07
Working status ^d^				2.92**	1.11	.09	3.25**	1.11	.10	3.17**	1.10	.10
Soc.distancing: 1/0 ^e^				–0.02	2.01	–.00	–0.80	1.98	.01	0.51	1.98	.01
Social distancing: 2/0 ^e^				0.44	1.18	.01	0.37	1.84	.04	1.16	1.16	.04
Health stressors ^f^							–2.49	1.93	–.04	3.94	3.16	.07
Financial stressors ^g^							–4.54***	1.07	–.14	–4.67***	1.07	–.15
Lockdown stressors ^h^							–2.59*	1.00	–.09	–1.92	1.03	–.06
Health stressors × Lockdown stressors										–10.20*	3.98	–.14
R-squared	.039			.074			.107			.114		
Adjusted R-squared	.034			.065			.095			.101		
∆R-squared	.039			.036**			.033**			.007*		
F-statistic (df)	8.84 (4;875)***			7.76 (9;870)***			8.67 (12;867)***			8.56 (13;866)***		
RMSE	14.4969			14.2266			13.9724			13.9198		

*Note. **a **0 *= *male, 1 *= *female. **b **0 *= *subsistence level, 1 = middle level, 2 *= *high level. **c **0 *= *no diagnosed conditions, 1 *= *have diagnosed conditions. **d **0 *= *non-working, 1 *= *working/employed. **e **0 *= *no self-isolation/distancing, 1 *= *strict self-isolation, 2 *= *reasonable distancing. **f **0 *= *no stressful situations 1 *= *faced illness. **g **0 *= *no stressful situations 1 *= *faced financial loss or job loss. **h **0 *= *no stressful situations 1 *= faced unavailability of goods or services. *p < .05. **p < .01. ***p < .001

Categorical variables with multiple categories were transformed into dummy variables. Three models were created with factors added sequentially to each model, while controlling the previous ones. The fourth model was the most complete due to the inclusion of the interaction of the variables; it was created to achieve maximum model fit.

The coefficients of determination show that all three groups of factors predict a similar proportion of the variance in the measured well-being (ΔR-squared model 1 = .039, ΔR-squared model 2 = .035, ΔR-squared model 3 = .033). However, the most complete model explains 11.4% of the variance in existential well-being. Adjusted coefficients of determination make it possible to compare models, since they consider the number of explanatory variables and the number of observations. We see that the fourth model, which considers the interaction of stressors, is the most accurate of the four presented models (Adjusted R-squared = .101).

The models allow us to estimate the significance of each factor. In the first group of factors, age and household monthly income were statistically significant. In the second group, working status was significantly positively related to well-being, while health conditions were negatively related. The negative effects of mental diseases were greater than those of physical diseases. Social distancing caused by the lockdown was not significant as a separate variable nor in its interaction with others. Problems caused by a job loss or a reduction in earnings and lockdown-related stressors were statistically significant.

Standardized regression coefficients allow us to compare the strength of the effect of each independent variable to the dependent variable. Based on the most complete model, the negative factors had the greatest effect: mental health status (*β* = –.16), financial stressors (*β* = –.15), and interaction of health stressors and lockdown stressors (*β* = –.14).

The interaction of variables was discovered by a combination of two stressors. The unavailability of goods or services moderated the connection between illness in the family and existential well-being. Increasing the moderator increased the effect of the predictor: having an ill family member did not produce a significant effect if the respondent could receive all the necessary assistance and medicines; but when these two stressors co-occurred, a significant decrease in well-being level was revealed.

ANOVA was used to determine whether the explanatory variables and their interactions were related to the dependent variable. The relevance of income appeared to be most prominent when comparing the difference between respondents who had a high household income and those whose income was close to the subsistence level (*F* = 11.186, *p* Tukey = < .001, Cohen’s *d* = 0.449). Respondents with higher income levels report a higher level of well-being, even during the pandemic. Upon comparison of groups by physical health status, no significant differences in well-being were observed (*F* = 0.354, *p* = .552, Cohen’s *d* = 0.043), while mental health had a greater effect (*F* = 28.465, *p* = < .001, Cohen’s *d* = 0.976). Working status was also a significant factor (*F* = 13.226, *p* = < .001, Cohen’s *d* = 0.267). The analysis of variance showed that being employed was associated with existential well-being, regardless of the respondent’s social status and income. Respondents who faced financial difficulties during the final week of lockdown more clearly demonstrated lower well-being (*F* = 24.183, *p* = < .001, Cohen’s *d* = –0.365).

## Discussion

### Assessing the existential well-being

The average of the Muscovites’ well-being during the lockdown was higher than the average TEM test scores as calculated for the general Russian population during an ordinary period. We assume either that initially high existential well-being in Moscow decreased during the pandemic but remained higher than in the whole of Russia, or that the existential well-being had not decreased at all. It is possible that the wellbeing of citizens has not declined due to changes brought about by the pandemic. Several other studies conducted in Russia at the beginning of the pandemic give further grounds for such an assumption.

Rasskazova et al. (2020) compared the well-being level between a group of 409 healthy adults in the period from April 17^th^ to April 26^th^ 2020, and three samples of 98, 66, and 293 people who completed the same tests (Satisfaction with Life Scale and Scale of Positive and Negative Experiences) in 2017 and 2019. Their results showed no differences between groups in the level of life satisfaction, although the intensity of positive emotions decreased. Some studies in other countries show similar data. The longitudinal study by Fernández-Abascal and Martín-Díaz (2021) comparing the level of well-being of Spanish adults throughout different weeks (a typical week, the week before the lockdown, and a week during the lockdown). They reviewed no progressive decrease of psychological well-being in either gender group over time. At the same time, the authors note that positive affects progressively decrease, while negative affects remain stable without increasing over time.

However, the results of global studies on well-being at the start of the pandemic remain conflicting. For example, Zhang et al. (2020) collected data on the well-being of 2231 adults living in 454 counties across 48 states in the US where the severity of the pandemic varied. The research was based on an analysis of Twitter profiles and tweets posted between April 1^st^ and April 24^th^. They found that pandemic severity gave rise to negative affects in adults (such as feeling scared, hostile, and nervous) rather than positive affects (such as excitement and enthusiasm), and the relationship between pandemic severity and the negative affects was moderated by personality and family connectedness. An Australian study by van Agteren et al. (2020), comparing the level of well-being (Satisfaction with Life Scale and MHC-SF), stress, and anxiety during the lockdown period, between March-April 2020, with the same indicators used from February 2019 to February 2020, showed that well-being and resilience were significantly lower during the period of the pandemic. In a study of Italian population stress and well-being during the pandemic, Rania & Coppola (2021) observed a decrease in well-being and mental health, regardless of gender differences and of whether or not participants had had direct contact with the virus.

We can see that the research results are not consistent due to the complexity of the phenomenon of well-being, a variety of measuring instruments, and the differences in lockdown conditions in different countries (and even within regions of one country). Thus, the conclusions about the greater or lesser significance of lockdown stressors cannot be universal.

If the level of well-being of Moscow citizens did not decrease, what could have determined its sustainability at the beginning of the pandemic? Under the existential approach, the absence of a decline may indicate the resistance of this form of well-being to situational changes. According to Längle (1993), existential fulfillment is the result of living with “inner consent”. During the measurement period, many residents had hope that the pandemic would recede in the summer and the stressors could seem like a challenge requiring a personal response. Lockdown created a new personal experience in many ways. Many residents began to pay more attention to their interests and communication with loved ones. These factors could support the inner consent and may have contributed to sustainability of existential well-being.

This result may also have occurred due to sample specifics. Moscow is a prosperous and wealthy city with an advanced social support system that had introduced additional support measures during the pandemic. It is possible that the citizen’s wellbeing in Moscow was higher before the pandemic and decreased under its influence but remained higher than in Russia as a whole. Clarification of this result provides an opportunity for future research.

Also, Pervichko et al. (2020) indicate that many Russians perceived COVID-19 as a “disease of the elite” at the beginning of pandemic. They believed that those affected were people who have opportunity to travel abroad (the entry route of the virus to Russia) and spend more time in informal communication, not limited by social distancing. The authors report that 38% of participants think the danger of COVID-19 is exaggerated.

Finally, the participants of the online studies can be assigned specific characteristics: they are socially active, well adapted to the online space and stay more connected to others. These factors are common for all online research (Payne & Barnfather, 2011), but during self-isolation, the opportunity to communicate online could significantly support the well-being of participants. However, clarifying the actual impact of these limitations requires testing additional hypotheses in future research.

### The effects of COVID-19-related stressors

We aimed to assess the impact of specific pandemic-related difficulties on Moscow citizens’ existential well-being. Ongoing living conditions and COVID-19-related stressors did not affect well-being as we expected. All groups of factors showed approximately equal statistical significance but had relatively weak explanatory power regarding existential well-being. Thus, both COVID-19-related stressors and ongoing living conditions predict well-being to a certain extent, but other factors were not measured in this study. This result emphasizes the importance of not neglecting both factors for predicting well-being: understanding the way a person is settled in life at a basic level is just as important as information about the difficulties that a person faced during the specific crisis.

Among the variables included in the group of long-term ongoing factors, the most significant was mental health. This result shows the crucial importance of taking a person’s mental state into account in well-being research. This finding is consistent with other studies, revealing that participants with self-reported mental health diagnoses had significantly higher distress, health anxiety, and fears of COVID-19 than those without a mental health diagnosis (Newby et al., 2020). However, given the small number (N = 31) of respondents diagnosed with mental conditions in our sample, this contrast should be treated with caution.

The most significant of the studied stressors were financial losses (a reduction in earnings or job loss) in the final week of lockdown and the co- occurrence of two stressors — illness and the unavailability of necessary services and medicines.

The importance of stable employment in times of change is shown. Similar results are discussed by Blustein and Guarino (2020): job loss provokes existential anxiety that has psychological consequences. Prime et al. (2020) emphasize that financial stability is one of the conditions for maintaining a safe living environment and therefore crucial for subjective well-being. From an existential-psychological perspective, support, a protected private space, reliability, and confidence in the future are prerequisites for well-being.

The discovered interaction of two stressors is interesting for the field of social welfare planning. Any physical illness that the respondent or someone in their family suffered from during the pandemic caused a decrease in well-being when social insecurity co-occurred with the instance of poor health. In such circumstances, growing stress can occur due to the unavailability of social services or the lack of access to necessary goods. Namely, Muscovites experienced temporal unavailability of free medical care for non-COVID-19 patients due to extreme congestion in hospitals. This combination of stressors is negatively related to existential well-being. This result can be used by social services to provide citizens with the necessary support.

## Conclusion

The study allows us to reconsider the impact of COVID-19-related stressors. In the context of the pandemic in Moscow during the first wave, we see a moderately high level of existential well-being and a moderate connection between existential well-being and COVID-related stressors when other factors are controlled. We may conclude that the impact of lockdown stressors is not universal. It varies according to region, living conditions, the severity of the lockdown, the dynamics of the pandemic, and cultural specifics.

The results may also vary depending on the measurement specifics of well-being. We assume that existential well-being can be resilient to rapid social changes, as it is more determined by internal factors like the ability to find meaning.

The existential well-being of the Moscow citizens during the first wave of the pandemic was affected by both the ongoing living conditions and COVID-19-related stressors (while sociodemographic variables were controlled). Thus, it is fruitful to use a comprehensive approach to measure the COVID-19-related stressors’ effect on well-being and is insufficient to consider only the frequency of exposure to stressful situations.

We discovered that a combination of COVID-19-related stressors (facing unavailability of goods, medicine, or services while falling ill or having a sick family member) was associated with poor well-being, while facing these situations separately did not produce a significant decline in well-being.

These results can find practical application in planning programs to support socially unprotected categories of citizens and in the work of social welfare services.

## Limitations

This research has been restricted by the unbalanced sample due to the research procedure (voluntary online survey), where female participants of the active middle age with access to the internet prevailed. It should also be noted that the research was carried out among Moscow citizens, thus the conclusions about the greater or lesser significance of lockdown stressors cannot be universal.

## Footnotes

1 100 000 RUB ≈ 1300 USD (for 2020–2021)
